# High-resolution 3D micro-CT reveals an additional muscle layer in esophageal atresia

**DOI:** 10.1007/s10388-026-01242-y

**Published:** 2026-08-31

**Authors:** Daniel Docter, Anastasia Ioannidou, Bernadette de Bakker, Joris J. T. H. Roelofs, Sjoerd de Beer, Sander Zwaveling, Jermo Hanemaaijer-van der Veer, Jon Sporring, Joep Derikx, Matthijs Oomen, Michiel van Wijk, Ramon Gorter

**Affiliations:** 1 Department of Paediatric Surgery, Emma Children’s Hospital, Amsterdam UMC, location AMC, Meibergdreef 9, 1105 AZ Amsterdam, The Netherlands; 2 Department of Obstetrics and Gynaecology, Amsterdam UMC, location AMC, Meibergdreef 9, 1105AZ Amsterdam, The Netherlands; 3 Amsterdam Reproduction and Development Research Institute, Meibergdreef 9, 1105AZ Amsterdam, The Netherlands; 4 Amsterdam Gastroenterology Endocrinology Metabolism Research Institute, Meibergdreef 9, 1105AZ Amsterdam, The Netherlands; 5 Department of Medical Biology, Amsterdam UMC, location AMC, Meibergdreef 15, 1105AZ Amsterdam, The Netherlands; 6 Erasmus MC – Sophia Children’s Hospital, Department of Paediatric Surgery, University Medical Center Rotterdam, Rotterdam, The Netherlands; 7 Department of Pathology, Amsterdam UMC, location AMC, Meibergdreef 9, 1105 AZ Amsterdam, The Netherlands; 8 Department of Computer Science, University of Copenhagen, Universitetsparken 1, 2100 København , Denmark; 9 Department of Paediatric Gastroenterology, Emma Children’s Hospital, Amsterdam UMC, Meibergdreef 9, 1105 AZ Amsterdam, The Netherlands

**Keywords:** Micro-CT, Pediatric Surgery, Esophageal Atresia, Histology, Musculature

## Abstract

**Background:**

Esophageal atresia (EA) is a congenital malformation intrinsically associated with esophageal dysmotility. Its etiology is poorly understood and it is unclear why motility is better preserved in some patients than in others. Esophageal motility is a three-dimensional (3D) process orchestrated by microscale structures, but conventional histology offers two-dimensional insight in the organization of the esophageal wall, hindering the identification of subtle, but potentially relevant, microarchitectural abnormalities that may contribute to understanding of the etiology of EA and variability in motility patterns. We aimed to evaluate the 3D microscale architecture of the esophagus in EA patients and identify potential anomalies using microfocus computed tomography (micro-CT).

**Methods:**

Full thickness esophageal samples from the proximal and distal pouch of eleven neonates undergoing EA repair and three fetal control esophagus samples (gestational age: 24 weeks) were collected, contrast-enhanced, and imaged with high-resolution micro-CT (2–5 μm voxel size). Muscle layers were segmented using the machine learning software RootPainter and EA samples were compared with the fetal controls. Findings were validated by histology.

**Results:**

Micro-CT enabled clear visualization of epithelial, submucosal, vascular, and muscular structures in all samples. 3D reconstructions consistently revealed a previously unreported oblique muscle layer situated medial to the circular muscle layer in all EA specimens. This layer varied in prominence and was not recognizable on 2D histology alone. Fetal control samples displayed only the typical circular and longitudinal layers, with oblique muscle fibres confined to the stomach.

**Conclusion:**

Three-dimensional assessment of esophageal microarchitecture using micro-CT allows differentiation between epithelium, musculature and vascularization. Moreover, it facilitated the identification of an oblique muscle layer in proximal pouch and distal trachea-esophageal fistula specimens from EA patients. It remains to be studied whether these findings can explain the observed variation in motility patterns in EA patients.

## Introduction

All patients with esophageal atresia (EA) suffer from esohageal dysmotility in varying degrees [1–3]. These symptoms are multifactorial, and deciphering the underlying mechanisms of motility patterns may provide potential research targets for novel treatment options. Esophageal peristalsis is a complex process. Its effectiveness relies on coordinated circular muscle contraction to propel the bolus and longitudinal muscle shortening to reduce luminal resistance and facilitate transit. The musculature and innervation responsible for this motion is microscale in size, and typically requires microscope-based methods to be visualized. Yet, the motion that they produce is three-dimensional (3D) and thus their architecture should be visualized in 3D to be fully appreciated.

Previous histological studies of resected material have demonstrated abnormalities of the esophagus, showing muscle fibre distortion by fibrosis, an increased number of submucosal glands, and the presence of cartilage [4, 5]. While traditional histological methods are crucial for diagnosing these abnormalities, they lack the capacity to provide 3D information on these microscale structures. Comprehensive 3D understanding of structural anomalies in muscle fibre orientation and myenteric plexus microanatomy is therefore lacking. Such abnormalities may be distributed throughout the esophagus or confined to specific regions, and relevant insights into dysmotility may lie in their three-dimensional organization.

Microfocus Computed Tomography (micro-CT) is an X-ray based computed tomography method that enables non-destructive visualization of microscale structures [6]. Developed in parallel to clinical CT in the geological and material science fields, it was optimized to have a higher resolution scan quality, at the expense of higher radiation dose and longer scan times. As such, it is not suitable for in-vivo practice, but can create 3D reconstructions in the micrometre scale of surgical resection material [7, 8]. In this study, we aimed to overcome the limitations of two-dimensional histology by applying micro-CT imaging combined with AI-based segmentation to generate three-dimensional reconstructions of esophageal muscle architecture in EA, thereby improving understanding of EA etiology and factors contributing to dysmotility.

## Materials and methods

### Sample collection and preparation

Patients presenting with esophageal atresia received standard of care treatment, including esophageal repair. During this procedure, the proximal pouch was opened and slightly resected to create vital tissue margins. After ligation, the TEF is disconnected from the trachea and also slightly resected to ensure optimal vascular supply of the anastomosis. This creates a small tissue sample from the pouch and TEF, which are usually discarded. After informed consent from parents of eligible patients, both samples were collected when available. For control samples, three esophageal samples were derived from human fetal samples of 24 weeks’ gestation. These foetuses, from elective terminations of pregnancy, had no known pathologies and were considered healthy. The control samples included the esophagus, laryngeal complex, trachea and part of the stomach. The foetuses were derived from the Dutch Fetal Biobank, in accordance with Dutch legislation [9]. All samples were fixed overnight in 4% paraformaldehyde (PFA). To enhance contrast for imaging, tissues were subsequently stained with 3.75% B-Lugol for 24 h. Samples were then mounted in 1.5% agarose to prevent movement during scanning. The study was approved by the medical ethics committee of the Amsterdam UMC under METC 2024.0367 (clinical) and METC 2016.0285 (fetal).

### Micro-CT imaging

Micro-CT scans were performed using a GE Phoenix Nanotom M Tomographer (General Electric, Wunstorf, Germany) available at the ContrasT facility in Leuven, Belgium. As sample size varied, for larger samples the scan settings were optimized to capture the entire sample, with a slight reduction of resolution. If samples were long in a single dimension, multiple scans were performed and stitched together to capture the full sample without compromising resolution. Scan parameters can be found in Table 1. Acquisition images were reconstructed with Phoenix |Datos| rec, the micro-CT manufacturer recommended software.

**Table 1:** Micro-CT Scan Parameters (GE Phoenix Nanotom M)

Sample	Voltage (kV)	Current (µA)	Filter	Voxel size	Exposure time (s)	SDD	Projections	Scan time (hh: mm)	Averages
EA1Prox	70	150	0.2 Al	2.5	500	480	2300	1:17	3
EA2Prox	70	150	0.2 Al	2.5	500	500	1800	1:01	3
EA3Prox	65	195	0.2 Al	3	500	400	2600	1:26	3
EA3Dist	60	210	0.2 Al	3	1000	500	3 × 1500***	5:00	3
EA4Prox	60	175	0.2 Al	2.5	1000	560	2 × 1500***	3:20	3
EA5Prox	65	195	0.2 Al	3	500	373	2600	1:31	3
EA6Prox	50	169	-	2	1000	500	2500	2:54	3
EA6Dist	60	175	-	2.5	750	600	3000	2:30	3
EA7Dist	60	140	-	2	1000	443	3000	3:22	3
EA8Prox	60	210	0.1 Al	3	750	467	3000	2:31	3
EA8Dist	60	140	-	1.5	1500	500	2 × 2000***	6:40	3
EA9Prox	60	245	0.1 Al	3.5	750	454	3000	2:30	3
EA10Combi*	80	210	0.5 Al	4	750	600	2500	4:14	3
EA11Prox	50	169	-	2	750	430	3000	2:34	3
EA11Dist	60	245	-	3.5	750	600	3000	2:32	3
EA12Prox	60	245	-	3.5	750	600	3000	2:31	3
EA12Dist	60	175	-	2	750	440	3000	3:45	3
FE1	80	260	-	5	500	600	2200	1:14	3
FE2	80	260	-	5	500	600	2200	1:14	3
FE3	80	260	-	5	500	600	2200	1:14	3

*EA * Oesophageal Atresia, *Prox * Proximal, *Dist.* Distal, *SDD*  Source Detector Distance

*EA10 included both proximal and distal end within a surgical metal clip due to a long gap approach in this case

** Sample detector distance

***Samples that were elongated were scanned multiple times to capture the entire length of the sample

### Histology

After scanning, the tissue samples were rinsed of the contrast agent in phosphate buffered saline (PBS). Samples were then paraffin embedded in sagittal orientation and sectioned in 5 μm thick slices. These were subsequently stained with Haematoxylin and Eosin (H&E) and evaluated by a panel including a dedicated paediatric GI pathologist with > 20 years of experience, complimented by a paediatric gastroenterologist, paediatric surgeon and a micro-CT expert. Histology results were compared with micro-CT to verify correct structure identification on the micro-CT scan. Samples were considered normal if they matched the following descriptions:

*Epithelia*: Nonkeratinized stratified squamous epithelium in the esophagus and columnar epithelium in the stomach.

*Innervation*: Nerve bundles of varying size, and presence of ganglion cells without dysmorphic features. *Vasculature*: Arteries and veins present in varying sizes without abnormal features, other than typical surgically induced trauma (hematomas).

*Muscles*: Muscle fibres arranged in circular and/or longitudinal orientation in these respective layers, without signs of hypertrophy or atrophy.

*Submucosa*: Situated between the muscularis mucosae and circular muscle layer, containing innervation, vascularization and submucosal glands. No signs of sclerosis or cartilage.

Final interpretation of the histological examination was performed by the pediatric GI pathologist.

### Data analysis

Scans were studied to determine the presence of structures of interest, including vessels, nerves, musculature, glands and epithelium. To visualize and quantify the musculature in the micro-CT scan, segmentations were made of muscular structures present in the esophagus, including the circular muscle layer (CML), longitudinal muscle layer (LML) and the muscular mucosa (MM). The machine learning software RootPainter, a deep learning segmentation tool originally developed for plant root analysis, was adapted here for tissue segmentation [10]. RootPainter was trained to segment muscle tissue and did not distinguish between different layers. To verify accuracy of the RootPainter model, in 2 samples these muscle structures were manually segmented. Segmentations were viewed in 3D by our expert panel to determine muscle fibre orientation and differentiate the respective layers. Manual segmentation of the structures of interest using Amira averaged 82.5 h per sample. RootPainter segmentations were comparable to the manual segmentations, but took 15.5 h of segmentations per sample, including model training, segmentation refinement and muscle separation. Therefore, all subsequent samples were only segmented using RootPainter. These segmentations were then viewed in Avizo Amira 2024.2 to separate the segmented musculature into their respective layers (CML, LML and MM).

## Results

**Fig. 1:**
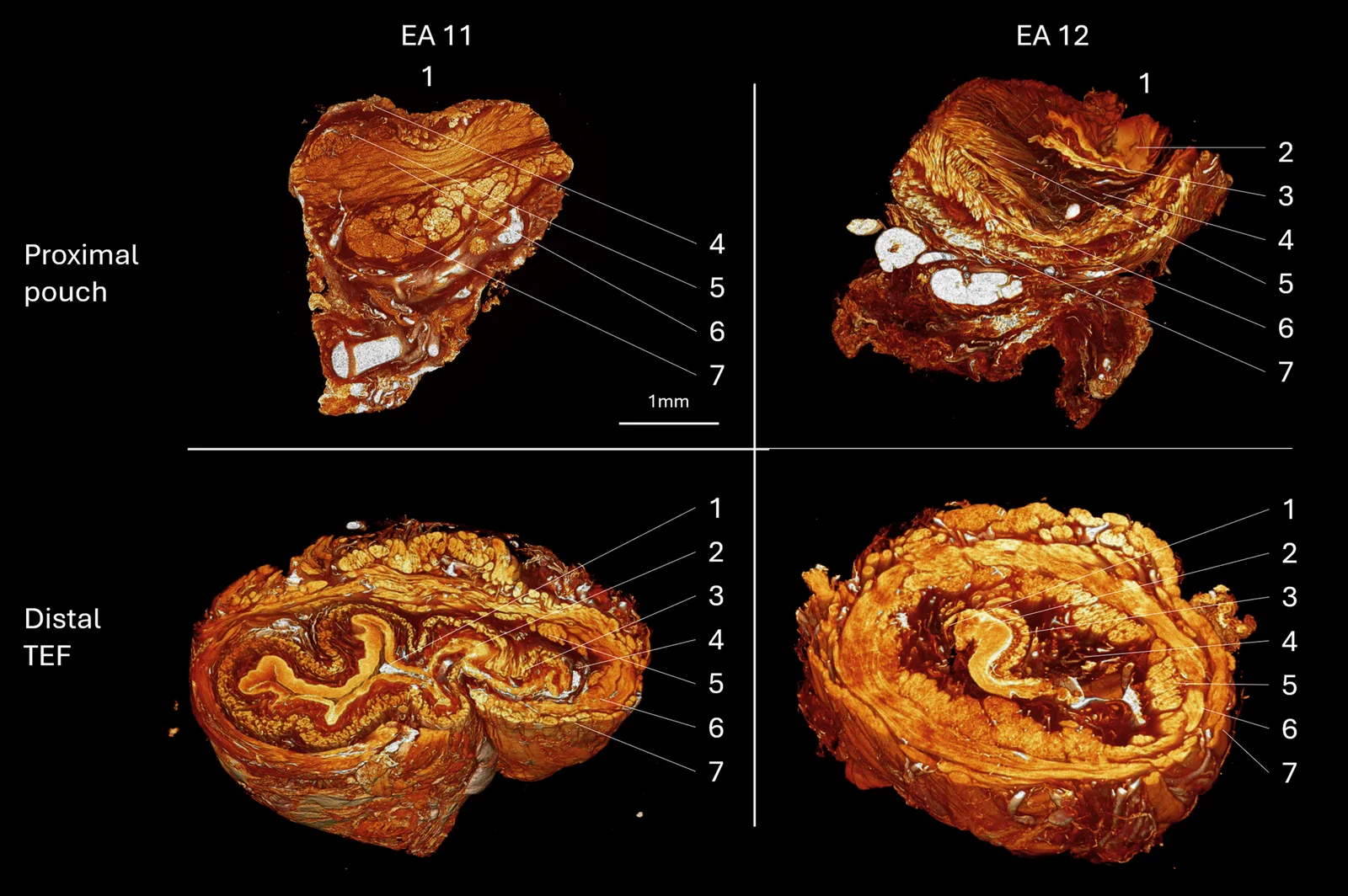
3D volume rendering with sagittal and axial cuts to reveal microstructures of EA11 and EA12. Notice how oesophageal layers can be distinguished and how an additional oblique muscle layer [5] is present in every sample. In EA12 distal, there appear to be 2 muscle layers on the left side in the image, that diverge into 2 muscle layers on the top side of the sample. (1) Lumen. (2) Epithelium. (3) Mucosa Muscularis. (4) Submucosa. (5) Oblique Muscle layer. (6) Circular muscle layer. (7) Longitudinal muscle layer

**Fig. 2:**
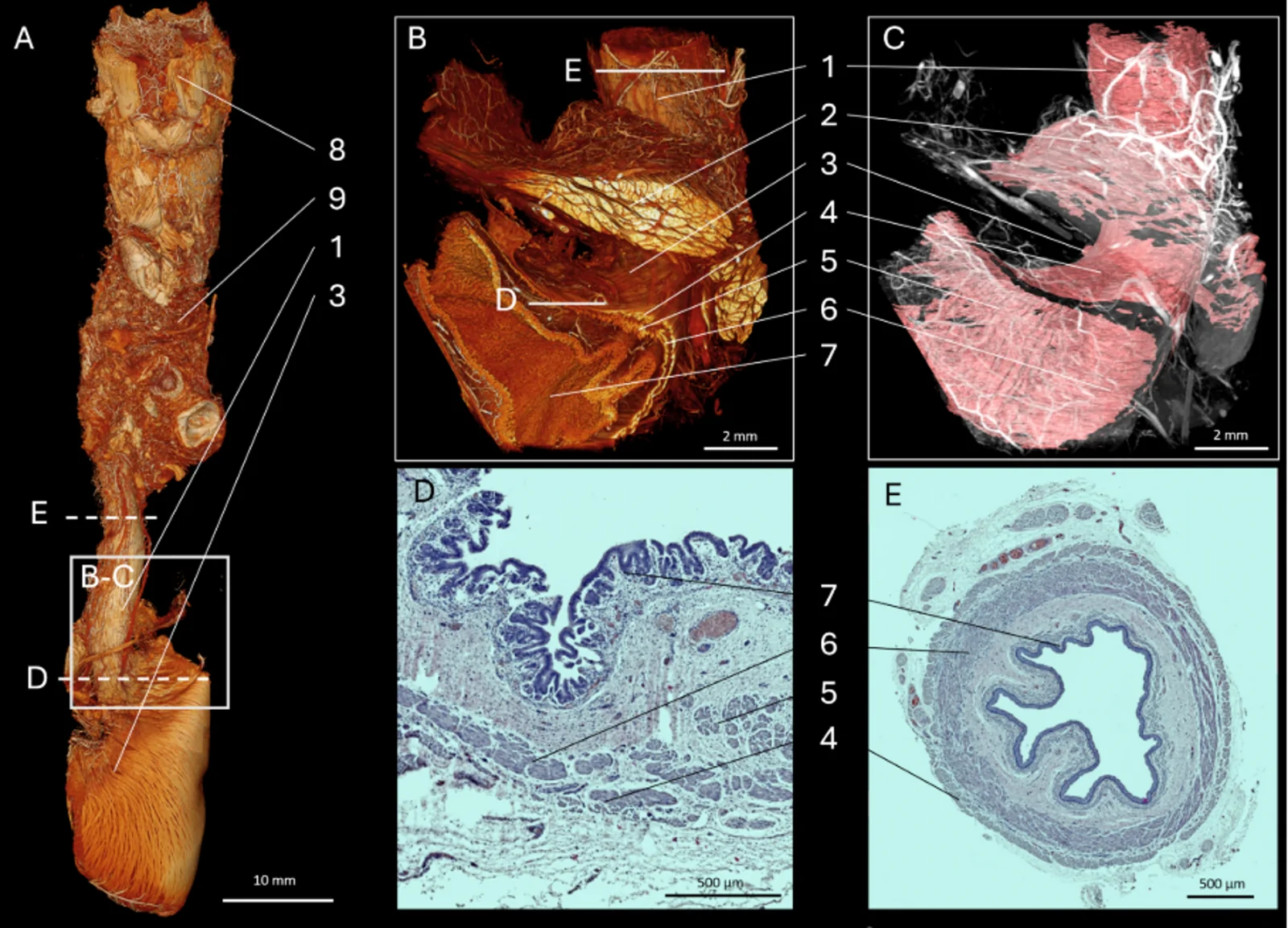
Micro-CT scan of a normal human fetal oesophagus and stomach. **A** 3D rendering of the entire sample. **B** Region of interest of the transition of the oesophagus towards the stomach. **C** Same region as B, showing a segmentation of the muscle layers enabling distinction of their muscle fibre orientation in either longitudinal, circular or oblique fashion. **D** Histological section of the same sample of the transition of the oesophagus into the stomach, showing 3 muscle layers [4, 6, 5]. **E** Histological section of the oesophagus, showing 2 muscle layers. (1) Oesophagus (2) Diaphragm (3) Stomach (4) Longitudinal muscle layer (5) Oblique muscle layer. (6) Circular muscle layer. (7) Epithelium (8) Larynx (9) Trachea

11 proximal samples and 7 distal samples were collected from 12 EA patients (11 Gross type C, 1 type B). Samples were not always available due to insufficient esophageal length, in which case there was not enough material for a sample. All collected samples were successfully scanned at a voxel size of 2–4 μm (Fig. 1). One sample (EA10) was derived from a patient with long gap EA and required esophageal approximation prior to anastomosis. This included clipping of the sample, which caused insurmountable artefacts on the scan and was therefore excluded. Demographic data of patients can be found in Table 2. For the fetal scans, overviews were performed of the entire sample at 10 μm, followed by a high-resolution detail scan of the gastro-esophageal junction at 5 μm **(**Fig. 2**)**.

**Table 2:** Demographic details

Inclusion	Age at time of surgery	Gestational Age at birth	Sex	Oesophageal atresia type	Other conditions of the patient
EA1	5 days	38 + 1	F	Type C	Heterozygote factor V Leiden
EA2	3 days	35 + 1	F	Type C	Persisting left Vena Cava Superior Tracheomalacia
EA3	7 weeks	29 + 0	M	Type B	-
EA4	2 days	38 + 3	M	Type C	Pulmonalis stenose
EA5	4 days	41 + 3	M	Type C	-
EA6	7 weeks	30 + 4	M	Type C	Ventricle septum defect Penoscrotal hypospadia Non testicular testis Inguinal Hernia Tracheomalacia Mosaic trisomy 16
EA7	10 days	39 + 3	F	Type C	Tracheomalacia
EA8	6 days	38 + 2	M	Type C	Tracheomalacia
EA9	5 days	37 + 6	M	Type C	-
EA10	8 days	37 + 4	M	Type C	Tracheomalacia
EA11	4 days	39 + 2	M	Type C	Tracheomalacia
EA12	5 days	37 + 1	M	Type C	Tracheomalacia
FE1	-	23 + 5	V	-	-
FE2	-	23 + 5	M	-	-
FE3	-	23 + 4	M	-	-

*EA * Oesophageal Atresia, *F* Female, *M* Male,

### Histology

**Fig. 3:**
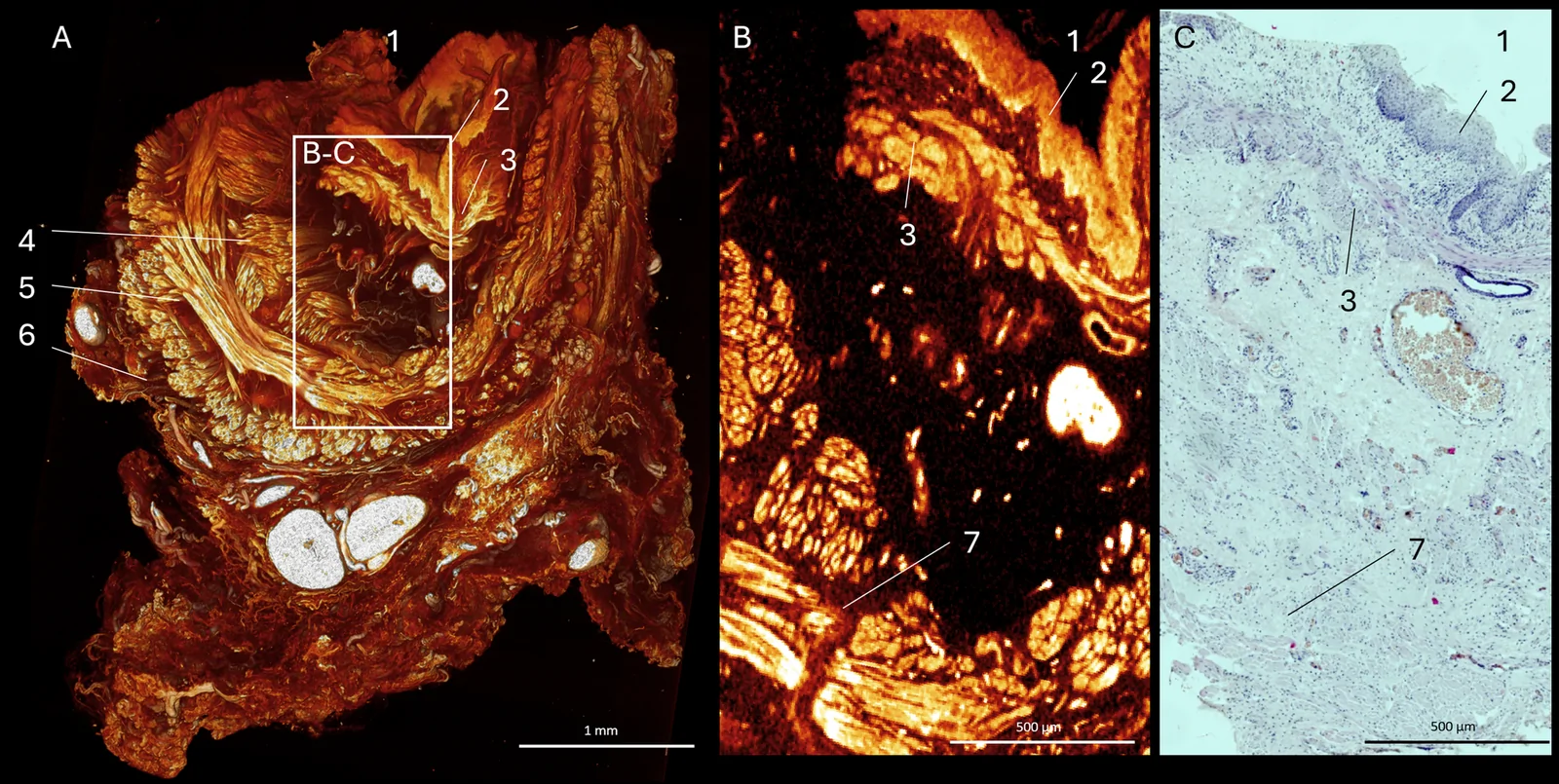
Micro-CT scan of a proximal oesophageal pouch. On the single slices of both the micro-CT **(Panel B)** and the histology **(Panel C)** the muscle layers of the oesophagus can be identified, but appear disorganized. Connective tissue can be seen between the fibres, giving the impression that they are compartmentalized but are not arranged in a typical longitudinal and circular fashion. in the 3D scan **(Panel A)** the layers can be seen more clearly, and a 3rd muscle layer becomes apparent with an oblique muscle fibre orientation. (1) Lumen (2) Epithelium (3) Mucosa muscularis (4) Oblique muscle layer (5) Circular muscle layer (6) Longitudinal muscle layer (7) Musculature (respective layer is unclear in 2D)

In all 16 included EA samples, smooth muscle was present. Layers could be distinguished with different muscle fibre orientation, namely the longitudinal and circular muscle layers. All musculature had a “compartmentalized” aspect, characterized by connective tissue being present within the muscle layers in every sample (Fig. 3). Nerves could be distinguished and innervation appeared normal in all but one sample (*n* = 15). In the abnormal sample (EA3 Prox) there was sparse innervation. Non-keratinized stratified squamous epithelium was present in 6 samples (3 proximal and 3 distal samples). No epithelium was present in the histological slices of the remaining 10 samples; therefore, it could not be interpreted. Results can be seen in Table 3. The additional muscle layer was not obvious from the histological slides. Aberrant muscle fibres could be seen in all samples, and gave a disorganized aspect to the musculature (Fig. 3). Fetal samples did not show this additional layer in the esophagus but had normal two-layered muscle anatomy in the esophagus, and three-layered musculature in the stomach.

**Table 3:** Histology and micro-CT analysis

Sample	Epithelium (Histology)	Epithelium (Micro-CT)	Musculature (Histology)	Musculature (Micro-CT)	Innervation (Histology)	Innervation (Micro-CT)	Vasculature (Histology/Micro-CT)	Submucosal glands (Histology/Micro-CT)
EA1 Prox	Normal	Normal	Disorganised	3- layer musculature	Normal	-	Normal	Normal
EA2 Prox	Normal	Normal	Disorganised	3- layer musculature	Normal	-	Normal	Normal
EA3 Prox	-	-	Disorganised	3- layer musculature	Sparsely present	-	Normal	Normal
EA3 Dist	-	Normal	Disorganised	3- layer musculature	Normal	-	Normal	Normal
EA4 Prox	-	-	Disorganised	3- layer musculature	Normal	-	Normal	Normal
EA5 Prox	-	Normal	Disorganised	3- layer musculature	Normal	-	Normal	Normal
EA6 Prox	-	Normal	Disorganised	3- layer musculature	Normal	-	Normal	Normal
EA6 Dist	Normal	Normal	Disorganised	3- layer musculature	Normal	-	Normal	Normal
EA7 Dist	-	-	Disorganised	3- layer musculature	Normal	-	Normal	Normal
EA8 Prox	-	-	Disorganised	3- layer musculature	Normal	-	Normal	Normal
EA8 Dist	-	Normal	Disorganised	3- layer musculature	Normal	-	Normal	Normal
EA9 Prox	-	-	Disorganised	3- layer musculature	Normal	-	Normal	Normal
EA10*	-	-	-	-	**-**	-	-	-
EA11 Prox	-	Normal	Disorganised	3- layer musculature	Normal	-	Normal	Normal
EA11 Dist	Normal	Normal	Disorganised	3- layer musculature	Normal	-	Normal	Normal
EA12 Prox	Normal	Normal	Disorganised	3- layer musculature	Normal	-	Normal	Normal
EA12 Dist	Normal	Normal	Disorganised	3- layer musculature	Normal	-	Normal	Normal
FE1	Normal	Normal	Normal	Normal	Normal	-	Normal	Normal
FE2	Normal	Normal	Normal	Normal	Normal	-	Normal	Normal
FE3	Normal	Normal	Normal	Normal	Normal	-	Normal	Normal

*EA *Oesophageal Atresia, *Prox*  Proximal, *Dist  *Distal

*EA10 was approximated, the sample contained both proximal and distal ends

### Micro-CT

**Fig. 4:**
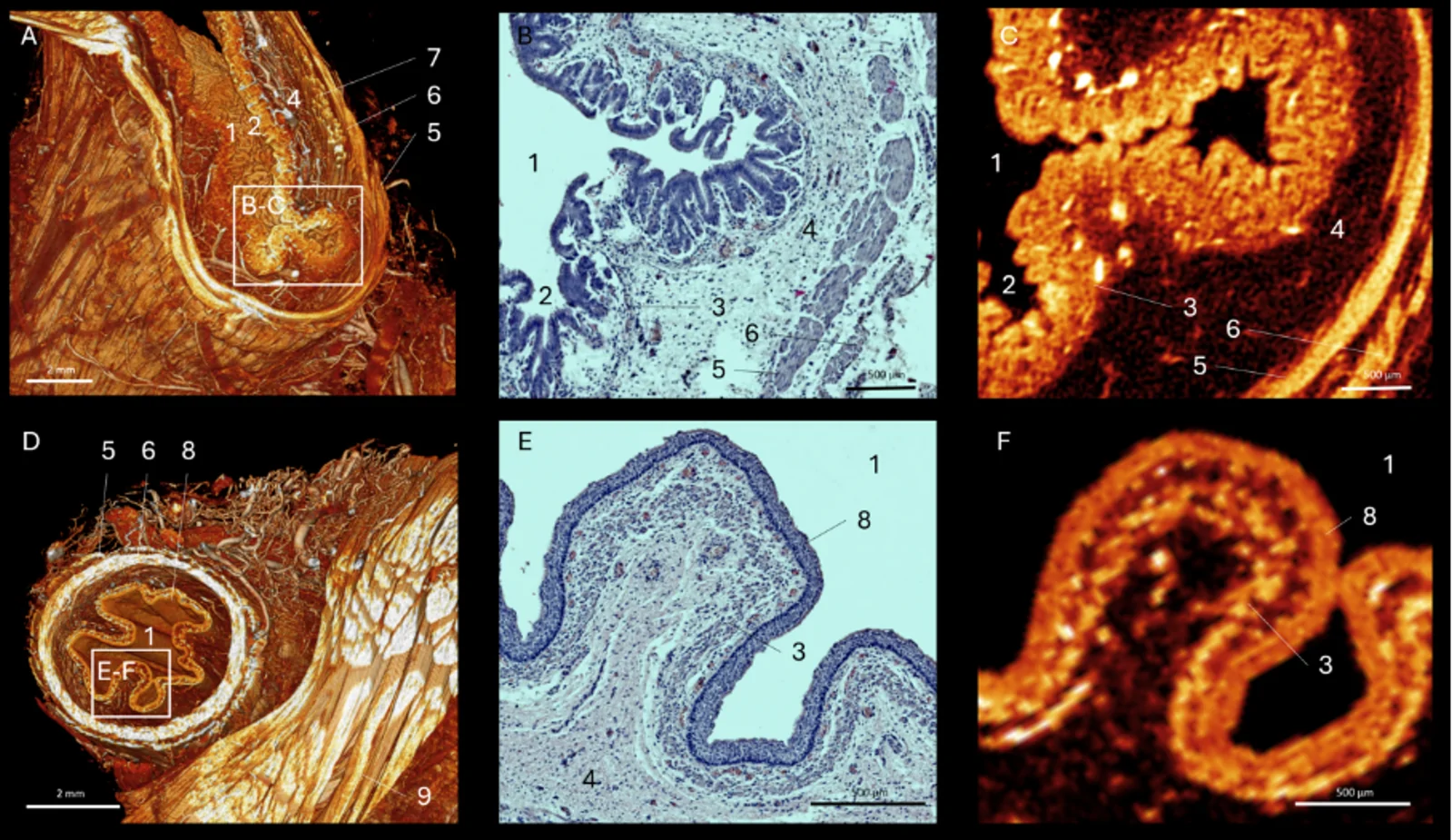
Comparison of epithelium between oesophagus and stomach. **A** 3D rendering of the micro-CT scan of FE1 showing the stomach at the cardia. **B** Histological slide of the epithelium of the stomach, showing columnar epithelium. **C.** Micro-CT scan at the same level as the histological slide, in which columnar epithelium can be recognized. **E** Histological slide of the epithelium of the oesophagus, showing Nonkeratinized stratified squamous epithelium **F.** Micro-CT scan at the same level, in which the same epithelium can be recognized. (1) Lumen (2) columnar epithelium (3) Mucosa muscularis (4) Submucosa (5) Circular Muscle layer (6) Longitudinal muscle layer. (7) Oblique muscle layer (8) Nonkeratinized stratified squamous epithelium (9) Diaphragm

**Fig. 5:**
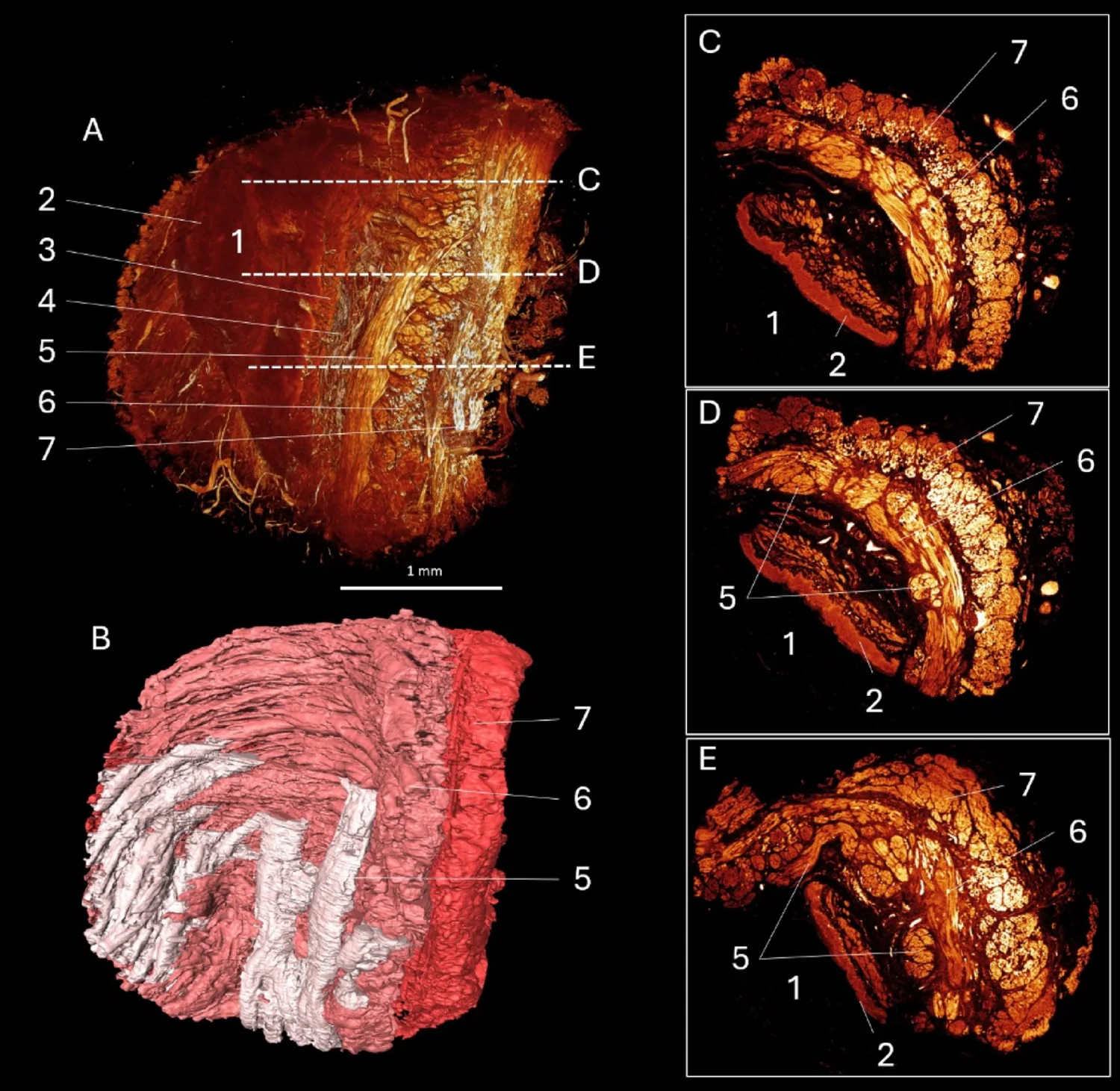
Possible evidence of the Gastro-Oesophageal junction in the proximal pouch of a patient. **A** Volume rendering of micro-CT scan of EA1, a proximal pouch of an oesophageal atresia patient. **B** Segmentation of muscle layers. Red: Longitudinal muscle layer. pink: Circular muscle layer. white: oblique muscle layer. **C** Axial section of the sample, with a normal buildup of muscle layers. **D** Axial section of the middle part of the sample, showing muscle fibres with an oblique orientation appearing within the circular muscle layer. **E** Axial slide of last part of the sample, showing a full oblique muscle layer. (1) Lumen. (2) Epithelium. (3) Mucosa Muscularis. (4) Submucosa. (5) Oblique Muscle layer. (6) Circular muscle layer. (7) Longitudinal muscle layer

Micro-CT scans enabled distinction between epithelium, submucosa, muscle layers and vasculature which could be correctly identified, in accordance with histology (Fig. 4**)**. Epithelium types could be distinguished in micro-CT, and this could be analysed in the entire sample. This allowed for identification of epithelium tissue in 5 additional samples, which was not present in the histological slices taken of those respective samples. All of these were nonkeratinized stratified squamous epithelium, fitting with normal esophageal epithelium. None of the EA samples showed signs of columnar epithelium. Nerves were more difficult to distinguish in micro-CT than histology, because of their poor B-Lugol binding and attenuation properties, thus they could not be interpretated on these scans. Additionally, micro-CT provided 3D data of the entire sample which allowed for muscle segmentation using RootPainter. These segmentations traced and grouped muscle fibres, which could be visualized in a 3D model and were interpretated by our expert panel **(**Fig. 5**)**. This led to the identification of a third muscle layer, obliquely oriented between the circular muscle layer and the submucosa (Fig. 1). This layer was present in every EA sample. In one sample (EA1 proximal), both a 2-layered and 3–3 layered musculature was observed. In this sample, the circular muscle layer appeared to divide in an oblique and circular layer. This phenomenon was not observed in other EA samples, which all (*N* = 15) had 3 muscle layers presented throughout the entire sample. In contrast, fetal control samples showed the typical circular and longitudinal muscle layer in the esophagus, with a third muscle layer present only in the stomach (Fig. 2).

## Discussion

This study demonstrates that micro-CT imaging, combined with segmentation analyses, can create 3D models of esophageal tissue which can provide novel insight. Using this technique, we identified a previously undescribed obliquely oriented muscle layer in EA esophageal specimens, which we did not observe in fetal control esophageal tissue. (Fig. 2**)**.

Within the GI tract, two muscle layers are typically present; an inner circular layer and an outer longitudinal layer, named according to the orientation of their muscle fibres. Under normal anatomical conditions, an additional muscle layer is only found in the stomach. At the gastro-esophageal junction (GEJ), the non-keratinized stratified squamous epithelium of the esophagus transitions into columnar epithelium of the stomach (Z-line). In this region, the circular layer divides into distinct circular and oblique layers. (Fig. 2) This oblique layer extends into the stomach up to the pylorus and contributes to mixing digestive fluids with food, enhancing motility and maintaining intragastric pressure [11, 12].

**Fig. 6:**
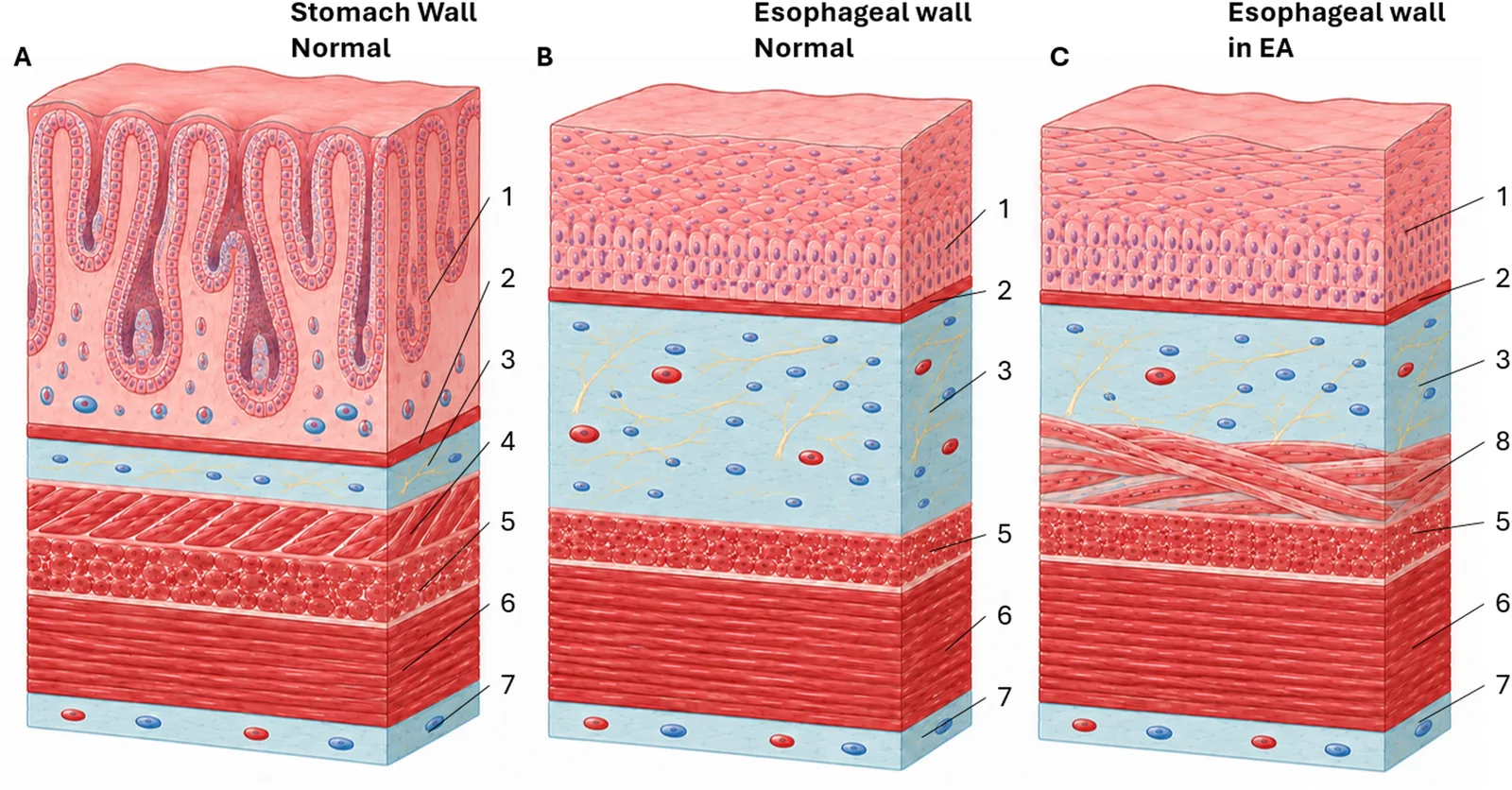
Illustrative schematic rendering of the different wall layers in the esophagus, stomach and esophageal atresia. Image was created with the help of AI and checked for accuracy by the authors. **A** The stomach has 4 muscular layers, the mucosa muscularis, the oblique muscle layer, the circular muscle layer and the longitudinal muscle layer. **B** in the esophagus, there is no oblique muscle layer. **C** In sample derived from children with EA, a chaotic muscle layer can be found between the circular muscle layer and the submucosa which consists of oblique oriented muscle fibered. (1) Epithelium. (2) Mucosa Muscularis. (3) Submucosa. (4) Oblique Muscle layer. (5) Circular muscle layer. (6) Longitudinal muscle layer. (7) Serosa (8) Chaotic oblique muscular layer

In esophageal atresia, previous histological studies have described abnormalities such as muscle fibre disorganization, fibrosis, and aberrant tissue components, but none have reported the presence of an additional oblique muscle layer [4, 13, 14]. The consistent identification of an oblique muscle layer in all EA samples in the present study is therefore notable. A plausible explanation lies in the three-dimensional nature of our imaging approach. Muscle disorganization is frequently described in EA, but its spatial arrangement may be difficult to interpret in two-dimensional histological sections. Three-dimensional visualization reveals that these seemingly disorganized fibres can form a coherent obliquely oriented layer. (Fig. 6).

The oblique muscle layer that was found in the EA samples was comprised of smooth muscle fibres, similar to the oblique muscle layer of the stomach. This was the case for both the proximal and distally acquired samples. The other muscle layers were also comprised of smooth muscle tissue. One would expect to find skeletal muscle in the proximal samples, as the esophagus most proximal third is comprised of this, and the middle third has a mixed muscle type [12, 15]. In previous histological studies of EA, although rare, pure smooth muscle was also found in the proximal derived samples. In most cases in that study however, the inner musculature was typically smooth and the outer musculature skeletal [5]. These discrepancies urge further research with larger sample sizes to determine muscle types in the esophagus of EA patients.

Given the small size of the tissue samples and the challenges associated with standardized embedding orientation, histological sections are likely to intersect these obliquely oriented fibres tangentially. This may result in misinterpretation or failure to recognize a distinct muscle layer. In the present study, the additional layer was not readily identifiable on histological sections alone and only became apparent upon comparison with micro-CT imaging (Fig. 3). These findings suggest that this layer may have been present in previously studied samples, but remained unrecognized due to the inherent limitations of conventional histology. In EA samples, the full mucosal structures may not be present which can make assessment challenging. Having the 3D scan, including the position of the obtained histological slide, may help to overcome this challenge and improve histological assessment in EA or similar samples.

Our findings raise the question of the extent to which this additional oblique musculature is present in patients with EA and its potential clinical relevance. Outside the stomach, an additional obliquely oriented muscle layer in the gastrointestinal tract has rarely been reported. To our knowledge, only two cases have been described in paediatric intestinal pseudo-obstruction, both involving the small intestine and identified by histology [16, 17]. Although small bowel motility is clearly abnormal in patients with pseudo-obstruction, it remains unclear whether the presence of this layer is related to the motility abnormalities seen. At present, there is no evidence to support a role for this oblique muscle layer in the etiology of esophageal motility abnormalities in patients with EA.

Moreover, it remains unclear whether this oblique muscle layer is present throughout the esophagus or limited to specific regions. In one proximal pouch, we identified a transition zone in which the proximal segment consisted of two muscle layers, while a third oblique muscle layer became apparent more distally (Fig. 4). This suggests that such transition zones may occur at different levels along the esophagus and could, in theory, contribute to the variability in motility abnormalities observed in patients with EA [3]. This raises the question whether resection of the esophageal section with an abnormal oblique layer in EA patients could have clinical impact on esophageal motility.

However, extending surgical resection margins may have significant risks and potential complications. Esophageal dysmotility in EA is likely multifactorial and may also be influenced by abnormalities of the enteric nervous system (ENS), which may be congenital or acquired during surgery. The current study suggests that the esophageal musculature in patients with EA is altered. Although innervation appeared normal in our histological slides, we were unable to map it in our micro-CT scans. The finding of an additional muscle layer raises questions in regards to this innervation, whether it has its own plexus, or how it reaches the oblique layer anatomically. Future studies should investigate the presence and extent of this oblique muscle layer, its innervational pattern, the existence of transition zones, and their relationship with esophageal motility before any clinical implications can be drawn.

The stomach-like appearance of the muscle layer in these samples might shed new light on the pathogenesis of esophageal atresia. One of the leading theories regarding EA etiology involves impaired neural crest cell migration, which has been proposed to underlie many of the associated anomalies that occur with esophageal atresia, including those seen in the VACTERL association [18, 19]. If neural crest–derived signalling pathways, that normally contribute to gastric development, are aberrantly present in the esophagus, this could influence muscle layer development and may also reflect altered development of the enteric nervous system. Such alterations in neural crest migration or signalling could potentially explain both the association of EA with other congenital anomalies and the presence of this additional muscle layer.

These findings warrant further research into genetical markers associated with gastric differentiation. For example, BARX1 is implicated in gastric development and in pathways involving neural crest–derived structures [20, 21]. In murine models, mutations in BARX1 have been associated with trachea-esophageal fistulas resembling those seen in humans [22]. To our knowledge, the role of BARX1 in EA has not yet been investigated in humans. If alterations in BARX1 or related signalling pathways are identified in EA, this would support a role for disrupted neural crest–related developmental mechanisms in the pathogenesis of esophageal atresia and explain the morphology that we observed here.

This study has several limitations. First, the sample size was small, reflecting the rarity of esophageal atresia, and paired proximal and distal samples were not available for all patients. Second, the tissue samples were limited in size (typically only a few millimetres), restricting assessment of longer esophageal segments. Ideally, larger segments would have been available to evaluate potential transition zones along the esophagus and to further explore this hypothesis. Finally, the control samples consisted of fetal tissue at 24 weeks’ gestation. Ideally healthy esophageal tissue from neonates would have been acquired, but this was not ethically possible within our institution. We therefore opted to utilize fetal tissue from the Dutch Fetal Biobank. Within Dutch law and legislation, the further developed fetuses were 24 weeks of gestation. Although relatively close in absolute age to term neonates, these samples are still undergoing maturation and therefore do not represent optimal controls.

## Conclusion

High-resolution micro-CT imaging combined with AI-based segmentation revealed the consistent presence of an additional muscle layer between the circular muscle and the submucosa in neonatal EA samples. These findings may contribute to a better understanding of the structural basis of dysmotility observed in patients with EA and highlight the value of advanced three-dimensional imaging techniques in the study of esophageal pathology. 

Moreover, the presences of a stomach-like morphology of the esophageal musculature in these patients can potentially shed new light on the etiology of esophageal atresia. Our study provides a foundation for future research into the developmental origins and functional implications of the structural abnormalities that we found. Ultimately, such insights may lead to the identification of treatment targets that may be beneficial for EA patients.

## Data Availability

Data can be made available upon reasonable request.

## References

[1] Lemoine C, Aspirot A, Le Henaff G, Piloquet H, Lévesque D, Faure C. Characterization of esophageal motility following esophageal atresia repair using high-resolution esophageal manometry. J Pediatr Gastroenterol Nutr. 2013;56(6):609–14. 10.1097/MPG.0b013e3182868773 23343933

[2] Courbette O, Omari T, Aspirot A, Faure C. Characterization of esophageal motility in children with operated esophageal atresia using high-resolution impedance manometry and pressure flow analysis. J Pediatr Gastroenterol Nutr. 2020;71(3):304–9. 10.1097/MPG.0000000000002806 32541198

[3] van Wijk M, Knüppe F, Omari T, de Jong J, Benninga M. Evaluation of gastroesophageal function and mechanisms underlying gastroesophageal reflux in infants and adults born with esophageal atresia. J Pediatr Surg. 2013;48(12):2496–505. 10.1016/j.jpedsurg.2013.07.024 24314193

[4] Al-Shraim MM, Ibrahim AH, Malki TA, Morad NA. Histopathologic profile of esophageal atresia and tracheoesophageal fistula. Annals Pediatr Surg. 2014;10(1):1–6.

[5] Dutta HK, Mathur M, Bhatnagar V. A histopathological study of esophageal atresia and tracheoesophageal fistula. J Pediatr Surg. 2000;35(3):438–41. 10.1016/s0022-3468(00)90209-4 10726684

[6] Docter D, Dawood Y, Jacobs K, Hagoort J, Oostra RJ, van den Hoff MJB, et al. Microfocus computed tomography for fetal postmortem imaging: an overview. Pediatr Radiol. 2023;53(4):632–9. 10.1007/s00247-022-05517-1 PMC10027643 36169668

[7] Docter D, de Bakker BS, Hagoort J, Roelofs J, de Beer S, van Heurn E, et al. Advancing Understanding of Anorectal Malformations Through Microfocus Computed Tomography Imaging of Resected Material. Gastro Hep Adv. 2025;4(5):100633. 10.1016/j.gastha.2025.100633 PMC12136826 40470484

[8] Docter D, Timmerman M, Dawood Y, Hagoort J, Lobe N, van Heurn E, et al. Scaling up contrast-enhanced micro-CT imaging: optimizing contrast and acquisition for large ex-vivo human samples. Forensic Imaging. 2024;37:200596.

[9] Dawood Y, Buijtendijk MFJ, Bohly D, Gunst QD, Docter D, Pajkrt E, et al. Human embryonic and fetal biobanking: establishing the Dutch Fetal Biobank and a framework for standardization. Dev Cell. 2023;58(24):2826–35. 10.1016/j.devcel.2023.11.019 38113849

[10] Smith AG, Han E, Petersen J, Olsen NAF, Giese C, Athmann M, et al. RootPainter: deep learning segmentation of biological images with corrective annotation. New Phytol. 2022;236(2):774–91. 10.1111/nph.18387 PMC9804377 35851958

[11] Birmingham A. The arrangement of the muscular fibres of the stomach. Trans R Acad Med Irel. 1898;16:432–40. PMC1327972 17232355

[12] Hur MS. Muscular architecture of the abdominal part of the esophagus and the stomach. Clin Anat. 2020;33(4):530–7. 10.1002/ca.23427 31301237

[13] Brosens E, Felix JF, Boerema-de Munck A, de Jong EM, Lodder EM, Swagemakers S, et al. Histological, immunohistochemical and transcriptomic characterization of human tracheoesophageal fistulas. PLoS ONE. 2020;15(11):e0242167. 10.1371/journal.pone.0242167 PMC7671559 33201890

[14] Merei J, Kotsios C, Hutson JM, Hasthorpe S. Histopathologic study of esophageal atresia and tracheoesophageal fistula in an animal model. J Pediatr Surg. 1997;32(1):12–4. 10.1016/s0022-3468(97)90081-6 9021557

[15] Kuo B, Urma D. Esophagus-anatomy and development. GI Motility online. 2006.

[16] Angkathunyakul N, Treepongkaruna S, Molagool S, Ruangwattanapaisarn N. Abnormal layering of muscularis propria as a cause of chronic intestinal pseudo-obstruction: A case report and literature review. World J Gastroenterol. 2015;21(22):7059–64. 10.3748/wjg.v21.i22.7059 PMC4462749 26078585

[17] Yamagiwa I, Ohta M, Obata K, Washio M. Intestinal pseudoobstruction in a neonate caused by idiopathic muscular hypertrophy of the entire small intestine. J Pediatr Surg. 1988;23(9):866–9. 10.1016/s0022-3468(88)80246-x 3183906

[18] Cozzi F, Myers NA, Piacenti S, Orfei P, Cozzi DA, Bonanni M, et al. Maturational dysautonomia and facial anomalies associated with esophageal atresia: support for neural crest involvement. J Pediatr Surg. 1993;28(6):798–801. 10.1016/0022-3468(93)90330-n 8331506

[19] Tovar JA. The neural crest in pediatric surgery. J Pediatr Surg. 2007;42(6):915–26. 10.1016/j.jpedsurg.2007.01.022 17560195

[20] Chin AM, Hill DR, Aurora M, Spence JR. Morphogenesis and maturation of the embryonic and postnatal intestine. Semin Cell Dev Biol. 2017;66:81–93. 10.1016/j.semcdb.2017.01.011 PMC5487846 28161556

[21] Stelzer G, Rosen N, Plaschkes I, Zimmerman S, Twik M, Fishilevich S, et al. The GeneCards Suite: From Gene Data Mining to Disease Genome Sequence Analyses. Curr Protoc Bioinf. 2016;54:1301–13. 10.1002/cpbi.5 27322403

[22] Woo J, Miletich I, Kim B-M, Sharpe PT, Shivdasani RA. Barx1-mediated inhibition of Wnt signaling in the mouse thoracic foregut controls tracheo-esophageal septation and epithelial differentiation. PLoS ONE. 2011;6(7):e22493. 10.1371/journal.pone.0022493 PMC3142160 21799872

